# NCAPG2 is a diagnostic biomarker associated with immune infiltration in cholangiocarcinoma

**DOI:** 10.1007/s12672-025-02809-4

**Published:** 2025-05-30

**Authors:** Zhaojun Huang, Xiaohang Niu, Mingjin Zhang, Xiaoming Wei

**Affiliations:** Department of General Surgery, The 901st Hospital of PLA, No.424 Changjiang West Road, Shushan District, Hefei, 230031 Anhui China

**Keywords:** NCAPG, Cholangiocarcinoma, Diagnostic, Immune infiltration

## Abstract

**Background:**

Cholangiocarcinoma (CHOL) is the second largest malignant tumor in the bile duct, only after primary liver cancer. Their high invasiveness can lead to poor prognosis. Over the past decade, research on CHOL has been ongoing, but there have been no breakthrough advancements. According to the literature, NCAPG2 is associated with the progression of various tumors, but its relevant role and value in CHOL have not been extensively studied.

**Methods:**

We studied the expression of NCAPG2 in CHOL and its clinical and pathological diagnostic value based on TCGA and GEO datasets. We evaluated the association between NCAPG2 and immune-related cells in CHOL using the TISIDB database.

**Results:**

We found that NCAPG2 has a higher expression level in CHOL and diagnostic significance in patients with CHOL. Additionally, we observed a correlation between immune cell infiltration and NCAPG2 levels in CHOL. Furthermore, NCAPG2 and its co-expressed genes were predominantly concentrated in ATP-dependent activity, DNA-related functions, and DNA helicase activity. The expression level of NCAPG2 is intricately linked to the pathological classification of CHOL.

**Conclusion:**

Current research indicates that NCAPG2 may provide effective assistance for the early diagnosis and pathological classification of CHOL, potentially regulating the immune microenvironment and thereby influencing the occurrence and progress of CHOL.

## Introduction

Cholangiocarcinoma is a type of cancer that originates from the epithelial cells of the bile duct, which poses significant clinical challenges due to its often late diagnosis and aggressive nature [1–3]. This disease not only inflicts considerable morbidity and mortality on affected individuals but also imposes a substantial economic burden on healthcare systems, particularly in regions with high prevalence rates [4, 5]. Current treatment modalities for CHOL primarily include surgical resection, liver transplantation, and palliative care, with chemotherapy and targeted therapies being employed in advanced stages [1]. However, these approaches are frequently limited by factors such as late-stage presentation, inherent resistance to conventional therapies of tumors, and the lack of early detected biomarkers [2]. As a result, there is an urgent requirement for novel diagnostic and treatment approaches aimed at enhancing patient results and bridging the considerable knowledge gaps in the pathogenesis and advancement of CHOL. This underscores the necessity of further research in this domain to enhance clinical management and therapeutic efficacy for CHOL.

The current investigation centers on the phenotypic effects associated with NCAPG2, a gene known to play a role in multiple cellular mechanisms, such as regulating the cell cycle and maintaining chromosomal stability [6, 7]. Previous research has established a relationship between NCAPG2 expression levels and tumorigenesis, particularly in cancers such as breast and colorectal cancer, where its overexpression has been associated with poor prognosis and aggressive disease phenotypes [8, 9]. Furthermore, NCAPG2 has been linked to the modulation of immune responses, suggesting its potential role in the tumor microenvironment [10, 11]. These associations underscore the significance of NCAPG2 in the pathophysiology of cancer, highlighting its potential for targeted therapy and biomarkers in disease progression, thereby emphasizing the innovative nature of our investigation into its functional roles and clinical implications.

In our research, we used a comprehensive bioinformatics approach utilizing data from The Cancer Genome Atlas (TCGA) to investigate the expression of NCAPG2 in CHOL and its potential diagnostic value. This methodology is advantageous as it allows for the integration of large-scale genomic data, facilitating the identification of biomarkers that may enhance diagnostic accuracy and therapeutic strategies. In this study, our main objective was to elucidate the role of NCAPG2 in CHOL, particularly its expression levels and their correlation with tumor immune response and methylation patterns. Through the examination of the relationship between the expression of NCAPG2 and several clinical factors, such as tumor staging and the infiltration of immune cells, we aim to provide insights into the potential of NCAPG2 as a diagnostic marker and its implications in tumor immunity.This research enhances the comprehension of the pathophysiology associated with CHOL and underscores the importance of NCAPG2 within the realm of cancer immunotherapy.

## Materials and methods

### Data acquisition

We obtained a dataset of mRNA expression that includes 44 samples of CHOL and 9 adjacent non-tumor samples, utilizing RNA sequencing with transcripts per million (TPM) metrics. This dataset was obtained from TCGA database (https://cancergenome.nih.gov) [12]. Through the GEO database (https://www.ncbi.nlm.nih.gov/geo/) [13], we accessed gene expression profiles of the GSE77984 dataset [14], which comprises 10 CHOL samples and 12 normal human cholangiocyte samples.

### Protein–protein interaction network

We obtained the data of protein–protein interactions from the STRING database (https://cn.string-db.org/) and constructed the protein–protein interaction network of genes co-expressed relate to NCAPG2 [15].An interaction was considered statistically significant if it had a combined score > 0.15. The Cytoscape 3.10.1 application was used to construct the network [16].

### Functional enrichment analysis

We conducted a thorough analysis to find the biological processes and pathways that NCAPG2 probably influence. We began by identifying 2,578 genes associated with NCAPG2 in CHOL using data from TCGA. Additionally, we found 286 genes related to survival outcomes in CHOL. These genes were then subjected to enrichment analysis through Gene Ontology(GO), which includes biological processes, cellular components, and molecular functions, as well as Kyoto Encyclopedia of Genes and Genomes(KEGG) pathway analyses.The Database for Annotation,Visualization, and Integrated Discovery (DAVID) was used to perform this analysis [17], and the R package was used to visualize the results. We set a significance threshold with a corrected p-value of less than 0.05 to ensure the reliability of our findings.

### Immune infiltration analysis

We used the single-sample gene set enrichment analysis (GSEA) method to perform the analysis of immune infiltration in CHOL samples. 24 different types of immune cell were analysed, and the relationships between CHOL and each specific immune cell subset were explored with Spearman’s correlation coefficient.

### LinkedOmics database analysis

We explored the expression profile of NCAPG2 by the LinkedOmics database (http://www.linkedomics.org). Furthermore, we conducted an analysis of GO and KEGG pathways related to NCAPG2. And we employed GSEA to analysed its co-expressed genes within the Link Interpreter module [18].

### TISIDB database analysis

To identify interactions between cancers and immune system, we used the TISIDB (http://cis.hku.hk/TISIDB/) [19]. To investigate the immunological importance of NCAPG2 in cancer, we explored the relationship between NCAPG2 methylation levels and immune checkpoint gene expression through the immune regulatory function of the TISIDB database. Furthermore, the correlation between the expressions of NCAPG2 methylation levels and chemokines along with their corresponding receptors was analyzed through the"chemokine"module.

### Statistical analysis

**Fig.1:**
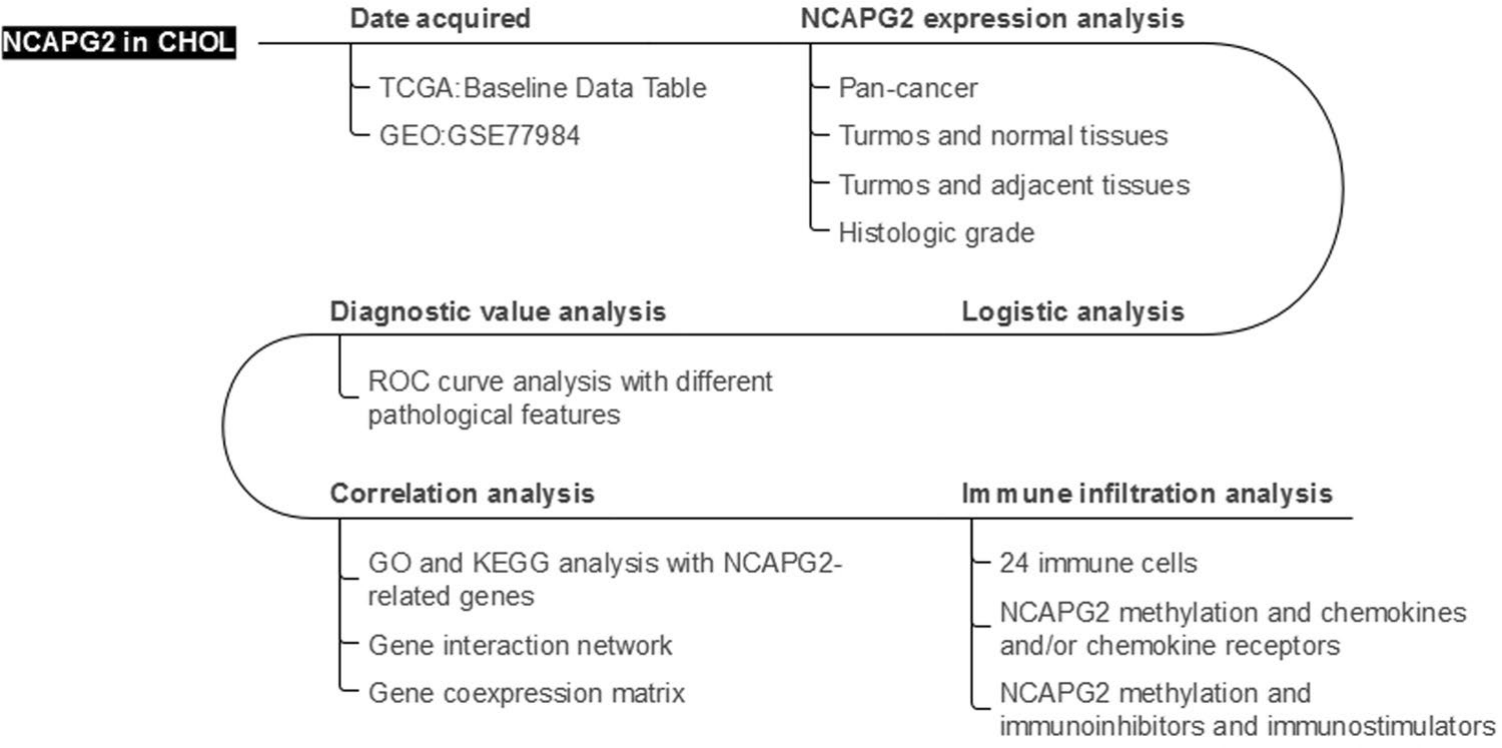
Workflow of the study

We used the box plots to evaluate the expression levels of the NCAPG2 in patients with CHOL. The median expression value of NCAPG2 served as the threshold for categorizing gene expression. The Wilcoxon signed-rank test and logistic regression analyses were used to investigate the relationship between NCAPG2 expression in CHOL and clinical characteristics. Furthermore, we conducted diagnostic receiver operating characteristic(ROC) analysis to evaluate the potential diagnostic utility of NCAPG2 expression, which also helped clarify its influence on other clinical parameters. All statistical analyses were performed using R statistical software (version4.2.1), and a p-value of less than 0.05 was deemed statistically significant. Figure 1 presents a workflow of the study.

## Results

### Patient baseline data sheet

A total of 35 patients diagnosed with (CHOL), along with their clinical characteristics, were obtained from the TCGA database. Table 1 details demographic and clinical characteristics. There were 19 females (54.3%) and 16 males (45.7%) in the study。In the study, a total of 35 patients were analyzed, with 17 individuals (48.5%) being 65 years old or younger, and 18 individuals (51.4%) being over the age of 65. According to CHOL stage, 18 patients (51.4%) in stage I, 9 (25.7%) in stage II, one (2.9%) in stage III and 7 (20%) in stage IV. Moreover, five patients (15.1%) had vascular invasion, while seven patients (21.8%) had perineural invasion. According to histological grade, one patient in grade 1 (2.9%), 15 in grade 2 (42.9%), 17 in grade 3 (47.6%) and two in grade 4 (5.8%).

**Table 1:** Clinical characteristics of patients with CHOL

Characteristics	Low expression of NCAPG2	High expression of NCAPG2	P value
n	17	18	
Pathologic stage, n (%)			0.657
Stage I	7 (20%)	11 (31.4%)	
Stage II	5 (14.3%)	4 (11.4%)	
Stage III	1 (2.9%)	0 (0%)	
Stage IV	4 (11.4%)	3 (8.6%)	
Pathologic T stage, n (%)			0.276
T1	7 (20%)	11 (31.4%)	
T2	6 (17.1%)	6 (17.1%)	
T3	4 (11.4%)	1 (2.9%)	
T4	0 (0%)	0 (0%)	
Pathologic N stage, n (%)			1.000
N0	12 (40%)	13 (43.3%)	
N1	2 (6.7%)	3 (10%)	
Pathologic M stage, n (%)			0.333
M0	12 (37.5%)	15 (46.9%)	
M1	4 (12.5%)	1 (3.1%)	
Gender, n (%)			0.505
Female	8 (22.9%)	11 (31.4%)	
Male	9 (25.7%)	7 (20%)	
Weight, n (%)			1.000
≤ 70	6 (17.1%)	6 (17.1%)	
> 70	11 (31.4%)	12 (34.3%)	
Age, n (%)			0.181
≤65	6 (17.1%)	11 (31.4%)	
> 65	11 (31.4%)	7 (20%)	
BMI, n (%)			0.457
≤25	6 (17.6%)	4 (11.8%)	
> 25	10 (29.4%)	14 (41.2%)	
Residual tumor, n (%)			0.645
R0	12 (37.5%)	15 (46.9%)	
R1	3 (9.4%)	2 (6.2%)	
Histologic grade, n (%)			0.082
G1	1 (2.9%)	0 (0%)	
G2	10 (28.6%)	5 (14.3%)	
G3	5 (14.3%)	12 (34.3%)	
G4	1 (2.9%)	1 (2.9%)	
CA19-9 level, n (%)			0.711
Normal	8 (27.6%)	5 (17.2%)	
Abnormal	8 (27.6%)	8 (27.6%)	
Vascular invasion, n (%)			0.335
No	15 (45.5%)	13 (39.4%)	
Yes	1 (3%)	4 (12.1%)	
Perineural invasion, n (%)			0.394
No	11 (34.4%)	14 (43.8%)	
Yes	5 (15.6%)	2 (6.2%)	
OS event, n (%)			1.000
Alive	8 (22.9%)	9 (25.7%)	
Dead	9 (25.7%)	9 (25.7%)	
DSS event, n (%)			1.000
No	9 (26.5%)	9 (26.5%)	
Yes	8 (23.5%)	8 (23.5%)	

*BMI* body mass index, *DSS* disease-specific survival, *OS* overall survival, *NCAPG2* non-SMC condensin II complex subunit G2

### The expression of NCAPG2 is upregulated in CHOL

**Fig. 2:**
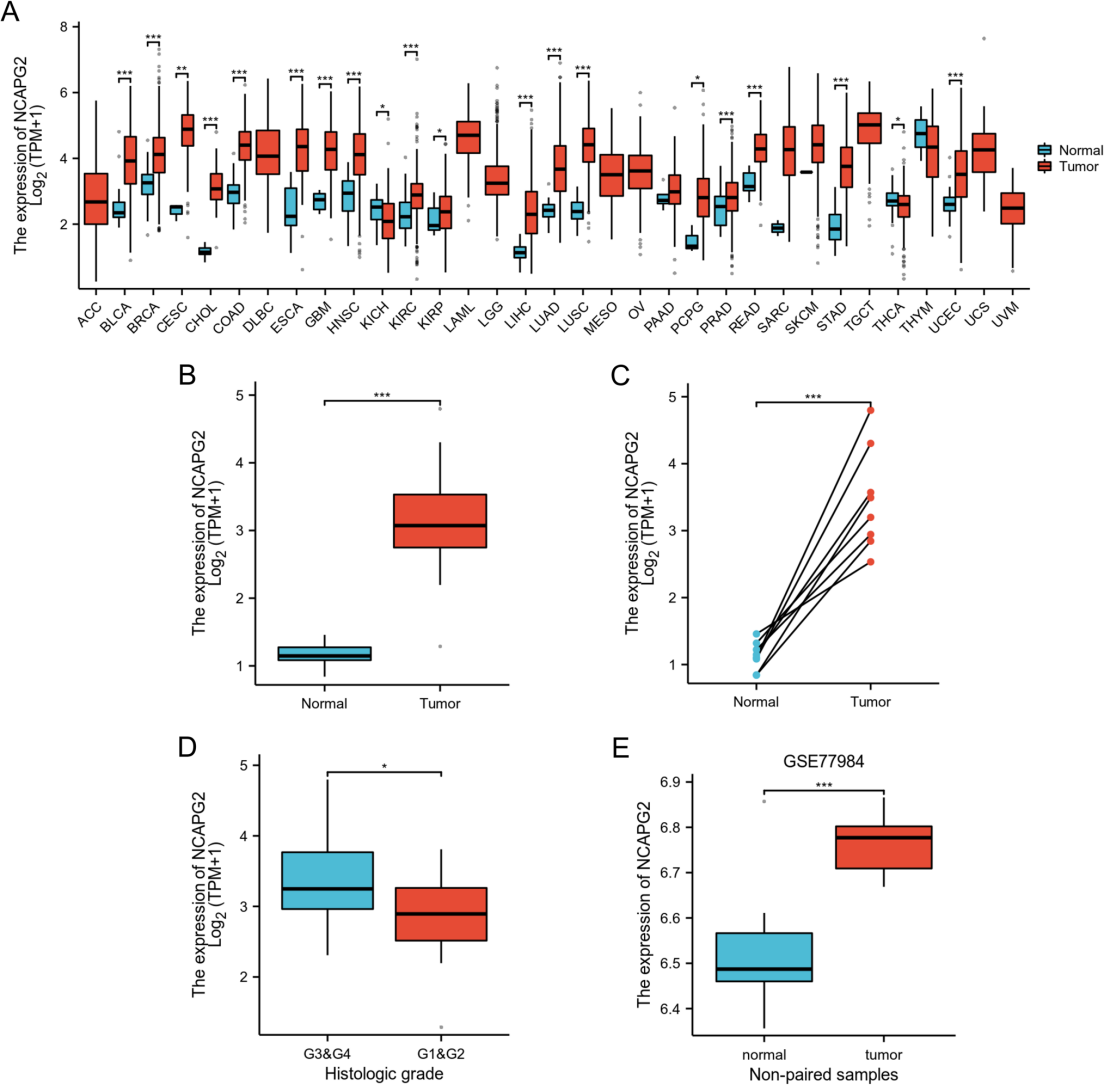
The expression level of NCAPG2 in different human cancers. **A** NCAPG2 expression in datasets from the TCGA database between multiple types of tumors and normal tissues. **B**–**D** NCAPG2 expression in normal tissues and paired adjacent tissues [unmatched tissues **B** and matched tissues (**C**)] and tumor tissues from patients with different clinical characteristics in TCGA [Histologic grade (**D**)]. **E** Expression of NCAPG2 in tumor and normal tissues in the GSE77984 datasets in the Gene Expression Omnibus (GEO) database.*p < 0.05, **p < 0.01, ***p < 0.001

To investigate the difference of NCAPG2 expression levels between normal and tumor tissues, the mRNA expression data for NCAPG2 was obtained from a variety of tumors and their corresponding normal tissues, as provided in the TCGA database. Then, we analysed its expression using the R programming language. The findings revealed a significant increase in the expression of NCAPG2 across a range of tumor tissues. This elevation was observed in various cancers, including CHOL, breast cancer, bladder urothelial carcinoma, hepatocellular carcinoma, lung adenocarcinoma and so on, when compared to their normal tissue counterparts (Fig. 2A). We compared the mRNA expression levels of NCAPG2 from tumor tissues with the adjacent non-tumor tissues. For normal tissues, our results indicated that the expression of NCAPG2 in CHOL was significantly higher. (p < 0.001; Fig. 2B). This increase was consistent in both CHOL and adjacent coupled cancer tissues (Fig. 2C). We analyzed the mRNA expression data of NCAPG2 across various clinical subcategories within the TCGA database. These results showed that elevated expression of NCAPG2 had an obviously correlation with histologic grade (p < 0.05) (Fig. 2D). Following this, we explored the relationship between different clinical parameters and patient prognosis using logistic regression analysis, which showed a link between histologic grade and overall survival (OS) (Table 2). These results suggest that NCAPG2 is notably upregulated in CHOL. To verify the expression profile of NCAPG2 in CHOL, we also examined an independent external dataset from GEO (GSE77984), focusing on the transcription levels of NCAPG2 in cancerous versus normal tissues. The results confirmed that NCAPG2 transcription levels in CHOL were obviously higher than in normal tissues (Fig. 2E). Overall, these findings support the conclusion that NCAPG2 is highly expressed in CHOL.

**Table 2:** Logistic analysis of the association between NCAPG2 expression and clinical characteristics

Characteristics	Total (N)	OR (95% CI)	P value
Pathologic T stage (T1&T2 vs. T3&T4)	35	5.231 (0.521–52.549)	0.160
Pathologic N stage (N0 vs. N1)	30	0.722 (0.102–5.095)	0.744
Pathologic M stage (M0 vs. M1)	32	5.000 (0.492–50.830)	0.174
Pathologic stage (Stage I&Stage II vs. Stage III&Stage IV)	35	2.083 (0.412–10.529)	0.375
CA19-9 level (Abnormal vs. Normal)	29	1.600 (0.362–7.073)	0.535
Gender (Male vs. Female)	35	0.566 (0.148–2.168)	0.406
BMI (> 25 vs. ≤25)	34	2.100 (0.467–9.440)	0.333
Age (> 65 vs. ≤ 65)	35	0.347 (0.088–1.371)	0.131
Weight (> 70 vs. ≤ 70)	35	1.091 (0.270–4.408)	0.903
Height (> = 170 vs. < 170)	34	0.385 (0.093–1.596)	0.188
Histologic grade (G1&G2 vs. G3&G4)	35	0.210 (0.050–0.879)	**0.033**
Child–Pugh grade (B vs. A)	20	0.800 (0.043–14.886)	0.881
Perineural invasion (Yes vs. No)	32	0.314 (0.051–1.940)	0.213
Vascular invasion (Yes vs. No)	33	4.615 (0.456–46.671)	0.195

### Diagnostic value of NCAPG2 expression in CHOL

**Fig. 3:**
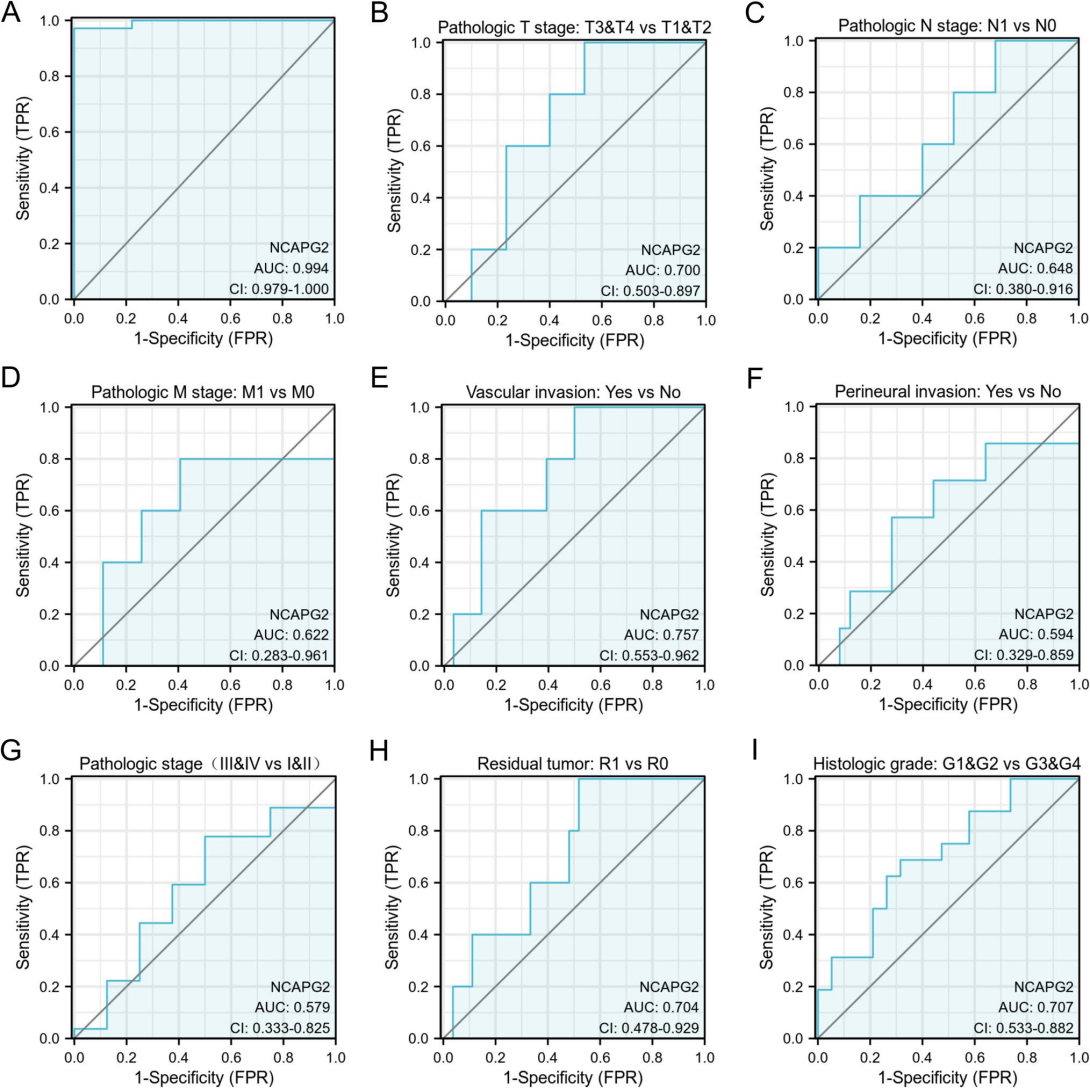
Diagnostic value of NCAPG2 expression in CHOL. **A** Receiver operating characteristic (ROC) curve analysis for NCAPG2 expression in CHOL and adjacent tissue. **B**–**I** Subgroup analysis for Pathologic stage T3&T4 and T1&T2 (**B**), Pathologic stage N1 and N0 (**C**), Pathologic stage M1 and M0 (**D**), Vascular invasion (**E**), Perineural invasion (**F**), Pathologic stage III&IV and I&II (**G**), residual tumor (**H**) and Histological grade (**I**)

We created a receiver operating characteristic (ROC) curve using NCAPG2 gene expression data sourced from TCGA database and assessed its diagnostic value in CHOL. The area under the ROC curve (AUC) was calculated to be 0.994, indicating a strong diagnostic capability, as shown in Fig. 3A. Additionally, subgroup analyses demonstrated that higher levels of NCAPG2 expression were significantly linked to improved diagnostic accuracy in CHOL across various parameters. Specifically, for the T stages (T1, T2, T3, and T4), the AUC was 0.700; for the N stages (N0 and N1), it was 0.648; for the pathological M stage, the AUC was 0.622; for vascular invasion, it was 0.757; for perineural invasion, it was 0.594; for pathological stages III and IV as well as I and II, the AUC was 0.579; for the presence of residual tumors, it was 0.704; and for histological grade, it was 0.707. These results are illustrated in Fig. 3B through 3I.

### Functional enrichment and analyses of NCAPG2-related genes in CHOL

**Fig. 4:**
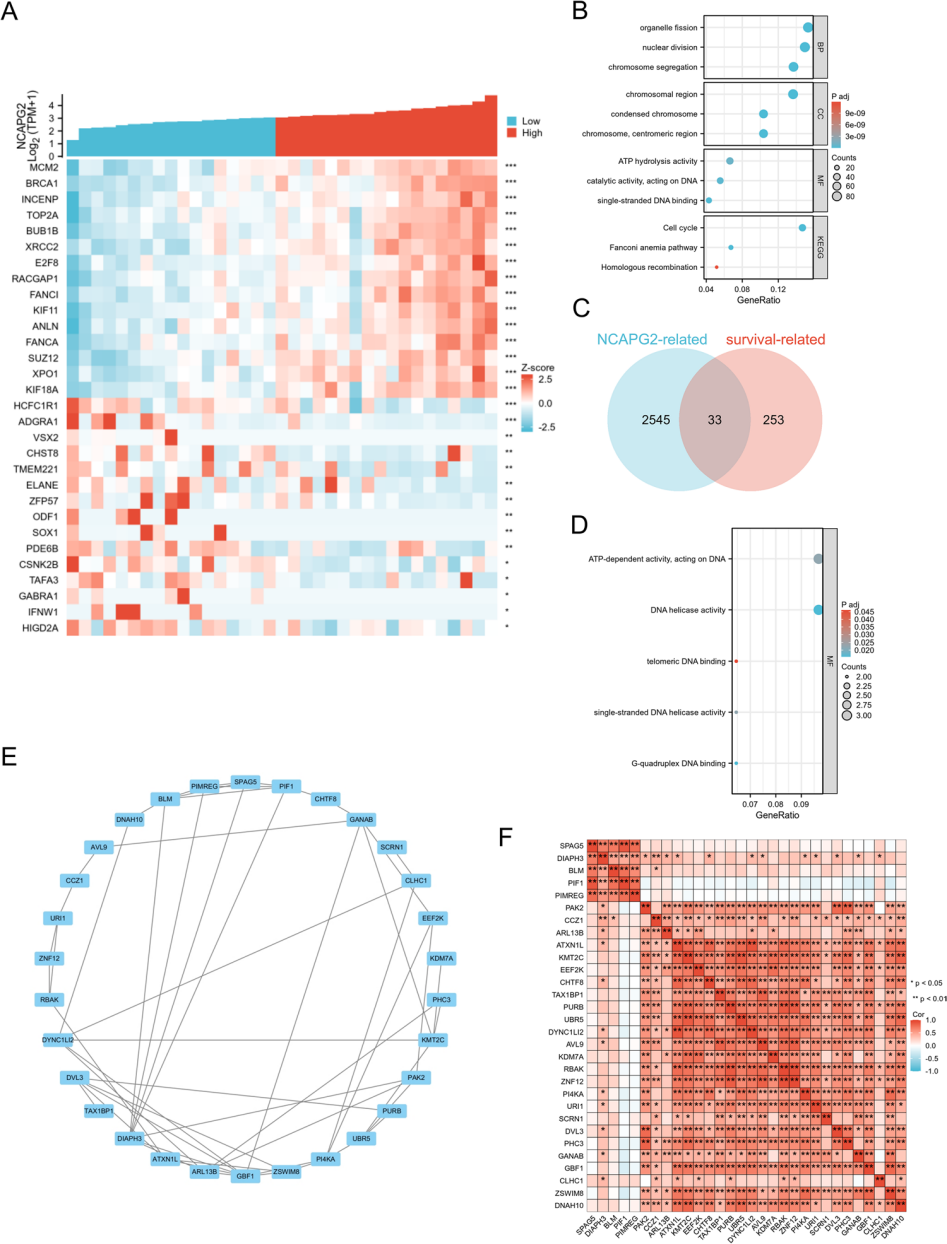
NCAPG2 functional clustering and interaction network analysis of NCAPG2-related genes. **A** Heatmap showed the positively top 30 genes with red and negatively top 30 genes with blue which related to NCAPG2. **B** GO and KEGG analyses of genes related to NCAPG2 in CHOL. **C** Venn diagram of upregulated genes related to NCAPG2 and survival in CHOL. **D** GO and KEGG analyses of genes related to NCAPG2 and survival in CHOL. **E** Interaction network of genes related to NCAPG2. **F** Gene co-expression matrix

In order to investigate the biological role of NCAPG2 in CHOL, we utilized the LinkFinder module available on the LinkedOmics platform to analyze the co-expression patterns of NCAPG2 within the TCGA database specific to CHOL. Our analysis revealed the top 15 genes that exhibited a positive correlation with NCAPG2, alongside the bottom 15 genes that displayed a negative correlation (illustrated in Fig. 4A). To further elucidate the functional implications of these NCAPG2-associated genes (top 600), we employed the DAVID Functional Annotation Bioinformatics Microarray Analysis tool to assess the enriched GO categories and the pathways derived from the KEGG. The findings revealed a notable enrichment in biological processes, including chromosome segregation, nuclear division, and organelle fission, associated with these genes (as shown in Fig. 4B and detailed in Table 3).To pinpoint genes exhibiting a concordant regulatory direction in patients with high NCAPG2 expression and poor survival outcomes, we intersected the 2578 genes (with an absolute value of correlation coefficient ≥ 0.4) that were most closely correlated with NCAPG2 and the 286 upregulated genes associated with survival (p Cox < 0.05) in CHOL. This analysis yielded 33 genes which are linked to NCAPG2 and survival in CHOL (depicted in Fig. 4C). These genes have the potential to serve as genetic biomarkers for patients diagnosed with CHOL. Subsequently, we conducted GO and KEGG analyses on these 33 genes, revealing that the genes were obviously enriched in ATP-dependent activities, particularly those acting on DNA, as well as in DNA helicase activity (illustrated in Fig. 4D and summarized in Table 4). Additionally, we performed analyses to investigate the interactions between proteins and their correlations, aiming to elucidate the relationships among these 33 proteins. Our findings revealed a significant enrichment network that includes these proteins (as shown in Fig. 4E). The analysis of gene co-expression correlations indicated that the majority of proteins within this network exhibited a strong positive relationship with one another, as illustrated in Fig. 4F. Therefore, it is evident that the genes linked to NCAPG2 show a considerable level of interconnectivity and could potentially function as multigene biomarkers for predicting survival outcomes in patients diagnosed with CHOL.

**Table 3:** Gene sets enriched in the NCAPG2-related genes (top 600)

Ontology	ID	Description	GeneRatio	BgRatio	P value	p.adjust
BP	GO:0007059	Chromosome segregation	76/555	348/18800	2.85e−44	1.09e−40
BP	GO:0000280	Nuclear division	83/555	446/18800	1.1e−42	2.1e−39
BP	GO:0048285	Organelle fission	85/555	493/18800	4.99e−41	6.36e−38
BP	GO:0098813	Nuclear chromosome segregation	66/555	287/18800	5.96e−40	5.7e−37
BP	GO:0000070	Mitotic sister chromatid segregation	51/555	171/18800	4.56e−37	3.49e−34
CC	GO:0098687	Chromosomal region	79/579	366/19594	1.65e−45	8.2e−43
CC	GO:0000775	Chromosome, centromeric region	60/579	227/19594	4.81e−40	1.2e−37
CC	GO:0000793	Condensed chromosome	60/579	255/19594	6.86e−37	1.14e−34
CC	GO:0000779	Condensed chromosome, centromeric region	45/579	156/19594	4.74e−32	5.89e−30
CC	GO:0000776	Kinetochore	42/579	146/19594	6.31e−30	6.27e−28
MF	GO:0003697	Single-stranded DNA binding	24/558	120/18410	1.6e−13	9.37e−11
MF	GO:0140097	Catalytic activity, acting on DNA	31/558	222/18410	1.25e−12	3.66e−10
MF	GO:0016887	ATP hydrolysis activity	37/558	325/18410	4.6e−12	8.98e−10
MF	GO:0008017	Microtubule binding	30/558	272/18410	1.09e−09	1.4e−07
MF	GO:0015631	Tubulin binding	36/558	376/18410	1.19e−09	1.4e−07
KEGG	hsa04110	Cell cycle	37/252	126/8164	8.81e−27	2.27e−24
KEGG	hsa03460	Fanconi anemia pathway	17/252	54/8164	2.08e−13	2.69e−11
KEGG	hsa03440	Homologous recombination	13/252	41/8164	1.38e−10	1.19e−08
KEGG	hsa04114	Oocyte meiosis	21/252	131/8164	4.19e−10	2.7e−08
KEGG	hsa03013	Nucleocytoplasmic transport	16/252	108/8164	1.69e−07	8.75e−06

*BP* biological process, *Bg* background, *CC* cellular component, *ES* enrichment score, *KEGG* Kyoto Encyclopedia of Genes and Genomes, *MF* molecular function; Gene sets with NOM p-value < 0.05 were considered as significantly enriched

**Table 4:** Gene sets enriched in the 33 genes at the intersection that were related to NCAPG2 and CHOL survival

Ontology	ID	Description	Gene ratio	BgRatio	P value	p.adjust
MF	GO:0051880	G-quadruplex DNA binding	2/31	10/18410	0.0001	0.0156
MF	GO:0003678	DNA helicase activity	3/31	72/18410	0.0002	0.0156
MF	GO:0017116	single-stranded DNA helicase activity	2/31	23/18410	0.0007	0.0223
MF	GO:0008094	ATP-dependent activity, acting on DNA	3/31	103/18410	0.0007	0.0223
MF	GO:0042162	telomeric DNA binding	2/31	37/18410	0.0018	0.0462

*MF* molecular function, *ES* enrichment score. Gene sets with NOM p-value < 0.05 were considered as significantly enriched

### Correlation between NCAPG2 expression and immune characteristics

**Fig. 5:**
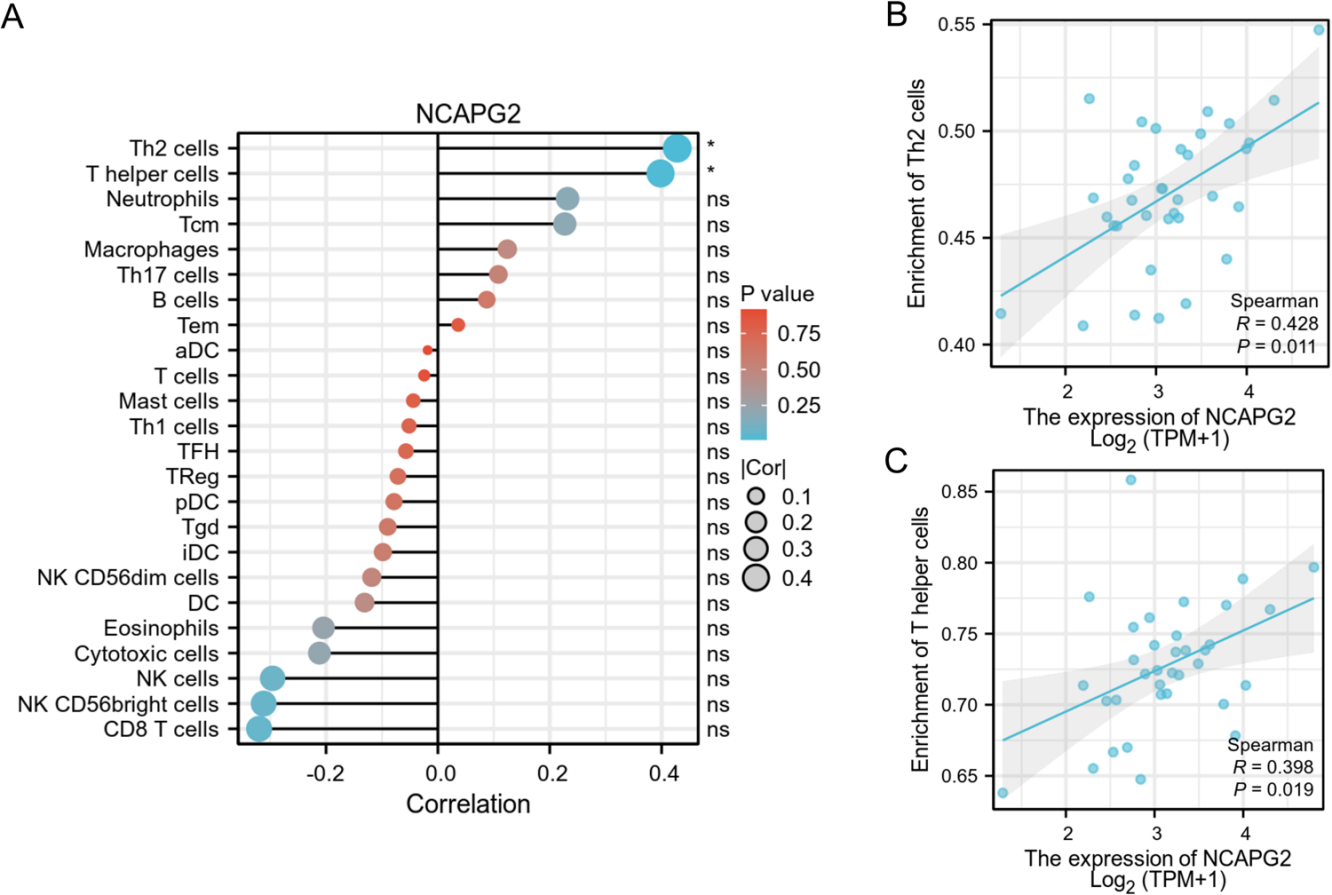
The relationship between NCAPG2 expression and immune infiltration in cancer. **A** Relative trend of 24 immune cells and NCAPG. **B**, **C** Th2 cell and T helper cell infiltration level related to NCAPG2

**Fig. 6:**
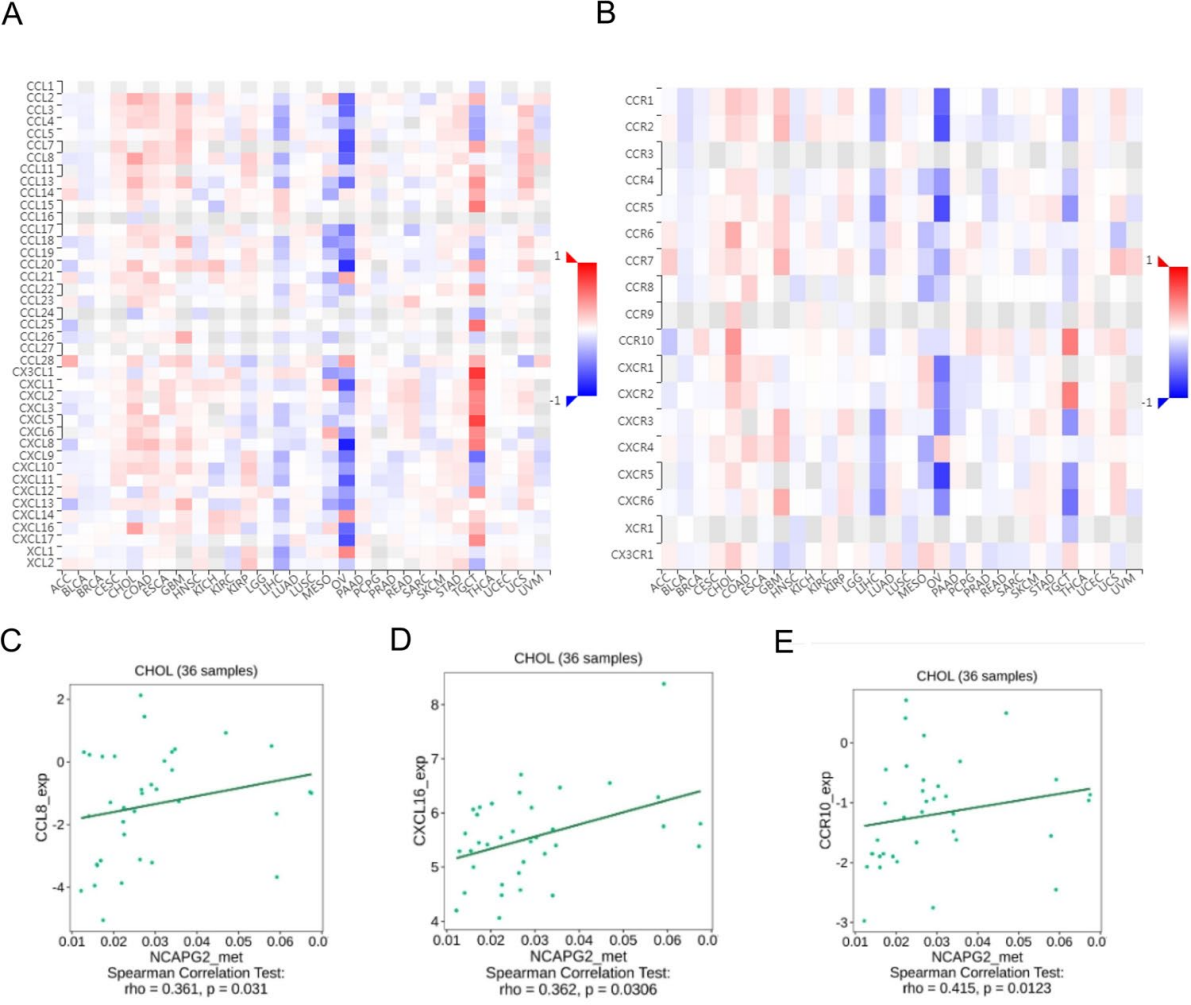
Relationship between NCAPG2 methylation and chemokines or their receptors. **A** Heatmap between NCAPG2 methylation and chemokines in cancers. **B** Heatmap between NCAPG2 methylation and chemokine receptors in cancers. (C–E) CCL8, CXCL16, CCR10 have a positive correlation with NCAPG2 methylation in CHOL

**Fig. 7:**
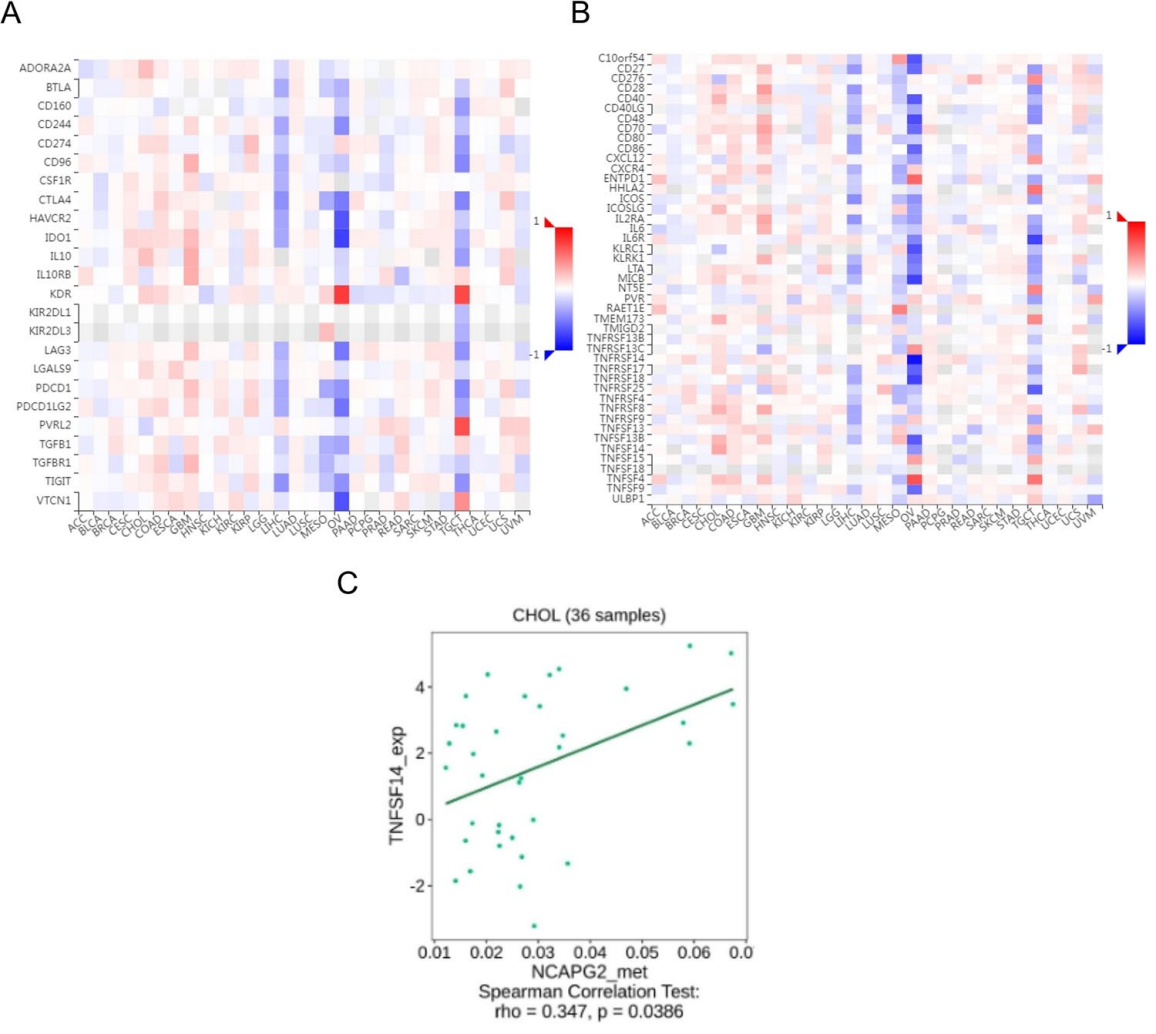
Relationship between NCAPG2 methylation and immunoinhibitors or immunostimulators. **A** Heatmap between NCAPG2 and immunoinhibitors in cancers. **B** Heatmap between NCAPG2 and and immunostimulators in cancers. **C** TNFSF14 has a positive correlation with NCAPG2 methylation in CHOL

We analyzed immune infiltration in CHOL using the TCGA database, focusing on how varying levels of NCAPG2 expression impacted this response. Our findings indicated that the expression of NCAPG2 is positively correlated with the presence of Th2 cells and T helper cells (Fig. 5A). A deeper examination of the relationship between NCAPG2 expression and immune cell infiltration in CHOL showed that higher NCAPG2 levels were positively associated with the infiltration of Th2 cells (r = 0.428, p = 0.011; Fig. 5B) and T helper cells (r = 0.398, p = 0.019; Fig. 5C). Additionally, to explore the link between NCAPG2 methylation and immune cell chemokines along with their respective receptors in CHOL, we utilized the TISIDB database. The resulting heat map demonstrated a correlation between NCAPG2 methylation levels and the presence of various chemokines and their receptors in CHOL (Fig. 6A, B). These results showed that NCAPG2 methylation may significantly influence tumor immunity. Then we further analyzed its relationship with chemokines and chemokine receptors, revealing a positive correlation between NCAPG2 methylation and the expression levels of CCL8 (r = 0.361, p = 0.031), CXCL16 (r = 0.362, p = 0.0306), and CCR10 (r = 0.361, p = 0.031; Fig. 6C–E). This indicates a positive relationship between NCAPG2 methylation and the expression of chemokines and their receptors in CHOL. Immune checkpoint inhibitors have emerged as a revolutionary approach in tumor immunotherapy, progressively improving the prognosis for patients with various cancer types. Therefore, we assessed the correlation between NCAPG2 methylation and the expression of immunoinhibitors and immunostimulators across different human cancers (Fig. 7A, B). Notably, NCAPG2 methylation showed a positive correlation with TNFSF4 (r = 0.347, p = 0.0386; Fig. 7C).Therefore, NCAPG2 may linked to regulate tumor immunity.

## Discussion

Cholangiocarcinoma is highly aggressive and heterogeneous resulting in poor prognosis [1, 2]. Pathologic features for prognosis included vascular invasion, tumor multiplicity, local extension, periductal infiltration and lymph nodal metastasis [20]. The diagnosis and prognosis of CHOL has not improved in the past decade. The development of CHOL is insidious [21]. Surgery remains the cornerstone of CHOL treatment [3, 22, 23]. For surgeons, early diagnostic markers are more needed. NCAPG2, a subunit of the chromosomal condensin II complex, is increasingly recognized for its role in tumor evolution [7–10]. Currently, there is very little research on NCAPG2 in CHOL. We utilized various bioinformatics methods to study the expression of NCAPG2 in CHOL and its potential regulatory network. According to this research, we assessed the expression levels of NCAPG2 across different types of cancer and then confirmed its elevated expression specifically in CHOL. Its high expression is related to the occurrence and pathological histological classification of CHOL, as well as vascular/nerve invasion, and has crucial value for the accurate diagnosis and pathological staging of CHOL. NCAPG2 can treat as an early diagnostic biomarker for CHOL, and its pathological diagnostic value can assist surgeons in better designing surgical plans before the operation. However, elevated NCAPG2 expression in cholangiocarcinoma diagnosis does not definitively exclude other malignancies, necessitating careful clinical consideration by surgeons.

To clarify the potential molecular mechanisms whereby‌ NCAPG2 affects the prognosis of CHOL, this study identified a total of 33 genes within the NCAPG2 gene network, which we designated as our primary focus. This includes a total of 286 genes that have a significant association with the prognosis of CHOL, along with 2,578 genes that show the strongest correlation with NCAPG2. We then performed a thorough analysis of the biological processes and signaling pathways linked to the key genes by utilizing GO functional enrichment and KEGG pathway analysis. The enrichment findings suggest that the majority of key genes are linked to biological processes including such as ATP-dependent activity and DNA helicase activity, which are essential for cellular processes such as DNA replication and repair. The Fanconi anemia pathway and homologous recombination, which is crucial for DNA repair and maintaining genomic stability, was also significantly associated with NCAPG2. This finding aligns with previous studies that have highlighted the importance of DNA repair mechanisms in cancer biology, where dysregulation can lead to genomic instability and tumorigenesis [24, 25]. Additionally, the pathways related to chromosome segregation and nuclear division are particularly relevant in the context of cancer biology [26–28]. Aberrant chromosome segregation can result in aneuploidy, contributing to tumor heterogeneity and resistance to therapy [26, 27]. The significant enrichment of NCAPG2-associated genes in these pathways suggests that NCAPG2 may play a critical role in ensuring proper mitotic processes, thereby influencing the overall survival and proliferation of CHOL cells. Further studies are warranted to elucidate the mechanisms underlying NCAPG2's role in CHOL.

Numerous studies have shown a significant link between cancer progression and the tumor microenvironment, which consists of immune cells, extracellular matrices, and inflammatory mediators. These components work together to promote tumor cell growth, survival, and spread [29, 30]. While a strong immune response can help fight tumors, cancer cells have developed ways to escape immune detection, such as impairing antigen presentation and attracting immunosuppressive cells. Previous research has indicated that the extent of immune cell infiltration in tumors can affect patient outcomes, with tumor-infiltrating lymphocytes being an independent prognostic factor for cancer patients [31, 32]. The current research showed the relationship between NCAPG2 expression and immune infiltration levels in CHOL.The findings indicated that the expression of NCAPG2 is positively correlated with Th2 cells and T helper cells. This aligns with previous studies that have highlighted the role of Th2 cells in promoting tumor progression through the secretion of cytokines that can enhance tumor growth and metastasis [33]. These results show that NCAPG2 may regulate the tumor immune microenvironment as well as in the advancement of CHOL.

Chemokines and their corresponding receptors are crucial in guiding the directional movement of immune cells. Chemokines play a crucial role in directing immune cell migration, and their dysregulation is often associated with poor prognosis in cancer [34, 35]. This research explored how the methylation status of NCAPG2 in CHOL correlates with the expression levels of chemokines and their receptors linked to immune cells, using data sourced from the TISIDB database. The findings revealed that the methylation status of NCAPG2 is positively correlated with the expression levels of CCL8, CXCL16, and CCR10. This suggests that NCAPG2 methylation might hinder the migration of immune cells into tumor microenvironment. Immune checkpoint blockade is an immunotherapy that has been applied in multiple tumors. TNFSF4 was explored to be closely related to breast carcinoma [36]. The positive correlation of NCAPG2 methylation with TNFSF4, an immunostimulatory molecule, further suggests that NCAPG2 may enhance the immune response against tumors, potentially serving as a therapeutic target for immune checkpoint inhibitors. This could elucidate the process through which NCAPG2 governs immune infiltration in CHOL.

In summary, we found that NCAPG2 is upregulated in CHOL and has significant value for early diagnosis and pathological classification, which can assist in surgical decision-making. At the same time, NCAPG2 can influence the progression of CHOL through immune infiltration.

## Limitation

This study still has limitations include the absence of functional validation, restricted cohort size and insufficient mechanistic explanation, which may introduce potential overestimation of clinical significance. These constraints highlight the need for multicenter validation and mechanistic investigations in future research. The next phase will include both in vivo and in vitro studies designed to clarify the role of NCAPG2 in CHOL, in order to enhance the reliability of the current research. Our research center will initiate the collection of cholangiocarcinoma specimens from recent years, with plans to conduct immunohistochemical analyses when resources permit. Our results indicate that NCAPG2 has strong diagnostic value for CHOL. But the observed AUC range indeed falls below the threshold for standalone clinical utility. Subsequent experiments will validate this through solid specimens and related tests of NCAPG2 in peripheral blood.

## Data Availability

All datasets used or analyzed in this study are accessible through publicly available databases.

## References

[1] Brindley PJ, et al. Cholangiocarcinoma. Nat Rev Dis Primers. 2021;7(1):65. 34504109 10.1038/s41572-021-00300-2 PMC9246479

[2] Banales JM, et al. Cholangiocarcinoma 2020: the next horizon in mechanisms and management. Nat Rev Gastroenterol Hepatol. 2020;17(9):557–88. 32606456 10.1038/s41575-020-0310-z PMC7447603

[3] Qurashi M, Vithayathil M, Khan SA. Epidemiology of cholangiocarcinoma. Eur J Surg Oncol. 2023;51:107064. 37709624 10.1016/j.ejso.2023.107064

[4] Parasuraman S, et al. Productivity loss outcomes and costs among patients with cholangiocarcinoma in the United States: an economic evaluation. J Med Econ. 2023;26(1):454–62. 36883994 10.1080/13696998.2023.2187604

[5] Darbà J, Marsà A. Analysis of hospital incidence and direct medical costs of intrahepatic cholangiocarcinoma in Spain (2000–2018). Expert Rev Pharmacoecon Outcomes Res. 2021;21(3):425–31. 33161795 10.1080/14737167.2021.1842201

[6] Zhang E, et al. NCAPG2 promotes prostate cancer malignancy and stemness via STAT3/c-MYC signaling. J Transl Med. 2024;22(1):12. 38166947 10.1186/s12967-023-04834-9 PMC10763290

[7] Li S, et al. Brachyury promotes proliferation and migration of hepatocellular carcinoma via facilitating the transcription of NCAPG2. Am J Cancer Res. 2022;12(8):3625–43. 36119840 PMC9442014

[8] Han S, et al. The mRNA stability of NCAPG2, a novel contributor to breast invasive carcinoma, is enhanced by the RNA-binding protein PCBP2. Cell Signal. 2023;110: 110844. 37544634 10.1016/j.cellsig.2023.110844

[9] Li R, et al. Mining and exploration of rehabilitation nursing targets for colorectal cancer. Aging (Albany NY). 2024;16(8):7022–42. 38637125 10.18632/aging.205739 PMC11087124

[10] Wang Q, et al. NCAPG2 could be an immunological and prognostic biomarker: from pan-cancer analysis to pancreatic cancer validation. Front Immunol. 2023;14:1097403. 36776838 10.3389/fimmu.2023.1097403 PMC9911455

[11] Chen X, et al. LncRNA-AL035458.2/hsa-miR-181a-5p axis-mediated high expression of NCAPG2 correlates with tumor immune infiltration and non-small cell lung cancer progression. Front Oncol. 2022;12:910437. 35664767 10.3389/fonc.2022.910437 PMC9160743

[12] Tomczak K, Czerwińska P, Wiznerowicz M. The Cancer Genome Atlas (TCGA): an immeasurable source of knowledge. Contemp Oncol (Pozn). 2015;19(1A):A68-77. 25691825 10.5114/wo.2014.47136 PMC4322527

[13] Clough E, Barrett T. The gene expression omnibus database. Methods Mol Biol. 2016;1418:93–110. 27008011 10.1007/978-1-4939-3578-9_5 PMC4944384

[14] Liao C, et al. microRNA-329 suppresses epithelial-to-mesenchymal transition and lymph node metastasis in bile duct cancer by inhibiting laminin subunit beta 3. J Cell Physiol. 2019;234(10):17786–99. 30887508 10.1002/jcp.28404

[15] von Mering C, et al. STRING: a database of predicted functional associations between proteins. Nucleic Acids Res. 2003;31(1):258–61. 12519996 10.1093/nar/gkg034 PMC165481

[16] Shannon P, et al. Cytoscape: a software environment for integrated models of biomolecular interaction networks. Genome Res. 2003;13(11):2498–504. 14597658 10.1101/gr.1239303 PMC403769

[17] Dennis GJ, et al. DAVID: database for annotation, visualization, and integrated discovery. Genome Biol. 2003;4(5):P3.12734009

[18] Vasaikar SV, et al. LinkedOmics: analyzing multi-omics data within and across 32 cancer types. Nucleic Acids Res. 2018;46(D1):D956–63. 29136207 10.1093/nar/gkx1090 PMC5753188

[19] Ru B, et al. TISIDB: an integrated repository portal for tumor-immune system interactions. Bioinformatics. 2019;35(20):4200–2. 30903160 10.1093/bioinformatics/btz210

[20] Ronnekleiv-Kelly SM, Pawlik TM. Staging of intrahepatic cholangiocarcinoma. Hepatobiliary Surg Nutr. 2017;6(1):35–43. 28261593 10.21037/hbsn.2016.10.02 PMC5332207

[21] Ohaegbulam KC, et al. The multidisciplinary management of cholangiocarcinoma. Cancer. 2023;129(2):184–214. 36382577 10.1002/cncr.34541

[22] Cillo U, et al. Surgery for cholangiocarcinoma. Liver Int. 2019;39:143–55. 30843343 10.1111/liv.14089 PMC6563077

[23] Kendall T, et al. Anatomical, histomorphological and molecular classification of cholangiocarcinoma. Liver Int. 2019;39(Suppl 1):7–18. 30882996 10.1111/liv.14093

[24] Rieckher M, et al. Distinct DNA repair mechanisms prevent formaldehyde toxicity during development, reproduction and aging. Nucleic Acids Res. 2024;52(14):8271–85. 38894680 10.1093/nar/gkae519 PMC11317141

[25] Uboveja A, Aird KM. Interplay between altered metabolism and DNA damage and repair in ovarian cancer. BioEssays. 2024;46(8): e2300166. 38873912 10.1002/bies.202300166 PMC11955923

[26] Mazzagatti A, Engel JL, Ly P. Boveri and beyond: chromothripsis and genomic instability from mitotic errors. Mol Cell. 2024;84(1):55–69. 38029753 10.1016/j.molcel.2023.11.002 PMC10842135

[27] Hosea R, et al. The two sides of chromosomal instability: drivers and brakes in cancer. Signal Transduct Target Ther. 2024;9(1):75. 38553459 10.1038/s41392-024-01767-7 PMC10980778

[28] Goh JJH, et al. Transcriptomics indicate nuclear division and cell adhesion not recapitulated in MCF7 and MCF10A compared to luminal A breast tumours. Sci Rep. 2022;12(1):20902. 36463288 10.1038/s41598-022-24511-z PMC9719475

[29] Xiao Y, Yu D. Tumor microenvironment as a therapeutic target in cancer. Pharmacol Ther. 2021;221: 107753. 33259885 10.1016/j.pharmthera.2020.107753 PMC8084948

[30] Hu G, et al. A bioinformatics approach to identify a disulfidptosis-related gene signature for prognostic implication in colon adenocarcinoma. Sci Rep. 2023;13(1):12403. 37524774 10.1038/s41598-023-39563-y PMC10390519

[31] Greten TF, et al. Immunology and immunotherapy of cholangiocarcinoma. Nat Rev Gastroenterol Hepatol. 2023;20(6):349–65. 36697706 10.1038/s41575-022-00741-4 PMC12468729

[32] Barkley D, et al. Cancer cell states recur across tumor types and form specific interactions with the tumor microenvironment. Nat Genet. 2022;54(8):1192–201. 35931863 10.1038/s41588-022-01141-9 PMC9886402

[33] Alam A, et al. Fungal mycobiome drives IL-33 secretion and type 2 immunity in pancreatic cancer. Cancer Cell. 2022;40(2):153-167.e11. 35120601 10.1016/j.ccell.2022.01.003 PMC8847236

[34] Ozga AJ, Chow MT, Luster AD. Chemokines and the immune response to cancer. Immunity. 2021;54(5):859–74. 33838745 10.1016/j.immuni.2021.01.012 PMC8434759

[35] Hozhabri H, et al. A comprehensive bioinformatics analysis to identify potential prognostic biomarkers among CC and CXC chemokines in breast cancer. Sci Rep. 2022;12(1):10374. 35725915 10.1038/s41598-022-14610-2 PMC9209453

[36] Li K, et al. The immunotherapy candidate TNFSF4 may help the induction of a promising immunological response in breast carcinomas. Sci Rep. 2021;11(1):18587. 34545132 10.1038/s41598-021-98131-4 PMC8452722

